# Development and preliminary validation of the Emotions while Learning an Instrument Scale (ELIS)

**DOI:** 10.1371/journal.pone.0255019

**Published:** 2021-08-27

**Authors:** Ingo Roden, Esther K. Friedrich, Sonja Etzler, Emily Frankenberg, Gunter Kreutz, Stephan Bongard

**Affiliations:** 1 Department of Educational Sciences, Institute of Education, University of Koblenz-Landau, Landau, Germany; 2 Department of Educational Psychology, Carl von Ossietzky University Oldenburg, Oldenburg, Germany; 3 Department of Psychology, Goethe-University Frankfurt, Frankfurt am Main, Germany; 4 Department of Music, Carl von Ossietzky University, Oldenburg, Germany; Universita degli Studi Europea di Roma, ITALY

## Abstract

Learning to play a musical instrument is associated with different, partially conflicting emotions. This paper describes the development and psychometric properties of the Emotions while Learning an Instrument Scale (ELIS). In a longitudinal study with 545 German elementary school children factorial structure and psychometric properties were evaluated. Exploratory and confirmatory factor analyses confirmed a two-factor solution measuring Positive musical Emotions while Learning an Instrument (PELI) and Negative Emotions while Learning an Instrument (NELI). Both subscales yielded scores with adequate internal reliability (Cronbach’s *α* = .74, .86) and relatively stable retest reliabilities over 18 months (*r* = .11 -.56). Preliminary evidence of congruent and divergent validity of the subscales is provided. Implications for future research of musical emotional experiences in children are discussed.

## Introduction

Previous research has shown that progress in learning to play a musical instrument in childhood is subject to a range of both extrinsic, e.g., parental support [1], and intrinsic influences, e.g., value beliefs [2], and self-regulation strategies [3]. This means that the failure or success of long-term music learning dependents on complex patterns of available resources and motivations [4–7]. Moreover, recent findings begin to highlight the importance of genetic factors in explaining the relationship between musical practice and ability [8,9].

One key implication is that musical practice in childhood is associated with both rewarding and stressful experiences of the learner [10]. In other words, music instrument learning may elicit strong positive and negative emotional responses, which could have predictive power to explain levels of commitment as well as long-term success or failure. However, at present there exists no psychometric inventory for the assessment of positive and negative affectivity in children who learn to play a musical instrument. Therefore, the present work was designed to fill this gap by developing a bi-dimensional inventory that can be applied to music education research, in general, and in the field of instrumental practice and learning, in particular.

### Social resources in learning to play musical instruments

The processes of learning and cognitive development in childhood are, in general, strongly affected by social relationships [11]. Therefore it is assumed that emotional experiences in learning musical instruments are also pertained by social aspects [12]. To address this issue in more depth [6] developed a questionnaire based on the Music Lesson Satisfaction Scale (MLSS; [13]) to investigate the triadic pupil-parent-teacher relationship in a cohort of eight to 18-year-old violin students including a wide range of proficiencies from beginners to experienced musicians. The authors observed an overall high level of enjoyment in students’ learning to play their instruments, but that receptiveness to parental support and pupil-teacher accord showed a significant influence beyond enjoyment, namely to affect the learners’ musical attainment, self-esteem, self-efficacy, motivation and satisfaction with music lessons. The authors conclude that the interpersonal dynamics amongst teachers, pupils, and parents influence learning outcomes, but also noted biases including the restriction to violin teaching and learning and other potential biases arising from the sampling strategy [6]. Further evidence for beneficial effects of musical learning on the social development of children with social disorders is reported from numerous studies in the field of music therapy [14–18].

The majority of studies examining the effects of musical learning on social skills, however, investigated the benefits of musical learning on self-regulation and self-efficacy. Meta-analytic research showed evidence, that self-regulation instruction improves academic performances and overall motivation [19] regardless of age or academic domain [20]. Similar results were confirmed in a systematic review by [21], in which the relationship between self-regulation and specific music learning variables of 25 quantitative, qualitative and mixed-methods studies had been examined. Participants’ ages varied from 7 to 45 years of age. Seven studies provide information about primary-school-age children [3,14,22–26]. In particular, the authors examined the relationship between music students’ self-regulatory characteristics and musical attainment, like the level of expertise and assessments of musical performance (e.g., exams, competition ect.), the amount of practice, the persistence during practice, the content of practice, ranging from informal practice, like improvisation and composition, to formal practice such as scales, techniques and teacher-selected repertoire, and the practice efficiency. Findings indicated weak, positive relationships between specific music learning and self-regulation behavior as well as for self-regulation instructions, suggesting the later as the most strongly related variable. However, none of the reported studies focused on the positive and negative emotional effects of musical activities in primary school children who just started to play a musical instrument.

### Positive and negative emotional effects of musical learning

Despite the increasing amount of research that was publish in the last 15 years on the relationship of music learning and self-regulation, self-efficacy, and motivation, the emotional effect of learning to play a musical instrument has only been the subject of very few studies [7,27–29].

For example, in a retrospective study Evans, McPherson and Davidson [7] examined the decisions to continue playing a musical instrument by analyzing data from primary school children who began learning a musical instrument 10 years prior to the study. When the data of their study were collected, the participants were between 18 and 20 years of age. Psychological needs like competence (feeling a sense of progress, enjoyment and pride in one’s progressing musical skills), relatedness (feeling socially connected and integrated through playing an instrument), and autonomy (feeling that one’s musical activities are self-endorsed and self-governed) were measured for the time when the participants were most engaged in playing and for the time when they stopped playing. Results showed, that decisions to cease playing an instrument were associated with weakened feelings of competence, relatedness, and autonomy, whereas feelings at the time when they were most engaged where not. Moreover, participants response to the reasons why they decided to cease playing, showed, that most participants referred to reasons that were related to denied feelings of psychological needs. In other words, those results showed, that the lack of positive emotions or the occurrence of negative emotions in the learning process could lead to the termination of playing a musical instrument. Thus, positive emotions on the one hand, as well as negative emotions on the other hand seem to play an important role in the process of learning a musical instrument.

Further evidence for the role of emotional needs and motivational aspects in music learning activities were reported in several studies by McPherson and Colleagues [2,3,27]. It was suggested, that motivational aspects were powerful predictors in children’s expectations of how long they thought, that they would learn their instrument. For example, McPherson [2] examined 133 primary school children between 7 to 9 years of age, who just started to learn a musical instrument. Results from interviews showed that the commitment to learn an instrument and the amount of practice was a good predictor for their instrumental performance after nine months of learning. It is of interest for the present study, that the observed children were able to distinguish between various motivational feelings, such as their interest in learning a musical instrument, the importance of being a good musician, and the cost-benefit ratio of learning a musical instrument. These findings suggest that the versatile motivational feelings of children might go hand in hand with positive and negative feelings of instrumental learning in primary school children.

In a more recent study, McPherson and colleagues [27] investigated additional emotional qualities and social factor that were immanent to a child’s decision, whether to continue or to cease to learn a musical instrument. The authors reported a significant influence of a) the sound, form and type of music learned during practice; b) whether a musical instrument was played in the family or in the peer group of the child; c) whether there was a positive support from family, teachers, and peers for practicing and playing an instrument; d) the enjoyment of playing an instrument; and e) the different self-regulation strategies that heled children to enhance their learning processes. Therefore, the development process of the questionnaire used in the present study included a variety of emotional qualities and social factors assess emotions while learning an instrument (see Table 2 for an overview of the items used in the ELIS).

A review article by Hallam [28] examined the active engagement with music on cognitive, social and personal development of children. For the social and personal development of children, the author reported evidence, that an increased amount of classroom music within the curriculum, had led to the increase of social cohesion within class, a greater self-reliance, as well as better social adjustment and more positive attitudes, specifically in disaffected pupils with low abilities. However, the author suggested, that the positive effects of the engagement with music on personal and social development strongly depends whether the engagement is perceived as an enjoyable and rewarding experience. Moreover, the personal or social benefits were directly related to the quality of the teaching and the extent to which individuals recognize that they are successful during the learning and playing process.

One problem with the reported results from a number of the cited studies must be considered with caution is, that most of them were based on self-reports and interviews. Hence, there is a need for standardized and validated questionnaires to assess the differential impact of musical activities on social and emotional behaviors.

When looking at the negative engagements of playing an instrument, the majority of the studies focused on the experiences of failure and performance anxiety. Both seemed to be relevant factors in the emotional experience of learning a musical instrument. For example, some studies showed, that musicians were burdened by psychological consequences of their performance anxiety or getting frustrated during practicing by being unable to get things right [29,30]. Moreover, performance anxiety does not seem to be present in professional performance only but also in rehearsals [31], and amateur performances [32]. Further, Ryan [33,34] showed, that children experience performance anxiety as much as adults do. According to a study by Simon and Martens [35], auditions alone, as well as auditions with a band, produce higher anxiety compared to athletic or academic challenges. Hence, as much as current research seems to point to positive emotional effects of learning an instrument, there are also accompanying negative effects that not only interfere with a child’s desire to continue playing an instrument but might even influence it`s general psychological wellbeing.

### Questionnaires and inventories of measuring positive and negative emotions of playing a musical instrument

The feasibility of studying the emotional impact of music training in child ren is highly dependent on the availability of a standardized test inventory. Chin and Rickard [36] developed the *Music Use Questionnaire (MUSE)* for assessing quality and quantity of different forms of music in two adult samples. Principal component analysis generated four reliable engagement styles: cognitive and emotional regulation, engaged production, social connection, as well as dance and physical exercise. It is of special interest for the present study, that the “cognitive and emotional” factor of the MUSE-Scale only includes positive aspects of music listening, e.g. “I often listen to music when I’m feeling down”, “Specific types of music make me feel better”, or “Music often takes away tension at the end of a day”. Also the engaged production or the social connection factor of the MUSE-Scale exclusively includes positive aspects of making music, like “Performing music is emotionally rewarding for me”, or “Being able to improve whilst playing music gives me a great sense of satisfaction”.

Creech & Hallam [6] investigated students’ perceptions of the pupil-teacher relationship in music educational settings of *N* = 347 violin students, whose average number of years of study was five. In particular, they examined how the interpersonal dimensions of *responsiveness* and *control* within pupil-teacher and pupil-parent relationships effects the process of learning a musical instrument. In addition, the researches were interested in how these variables influenced the pupils’ *enjoyment of music*, *satisfaction with violin lessons*, *motivation*, *self-efficacy*, *self-esteem* and *attainment*. The pupil’s *responsiveness* and *control* were assessed via the *Music Lesson Satisfaction Scale (MLSS)* by Rife, Shnek, Lauby & Lapidus [13], and the *Questionnaire on Teacher Interaction (QTI)* by Wubbels, Creton, Levy & Hooymayers [37]. The *MLSS* was adapted to measure pupils’ self-efficacy, self-esteem, enjoyment, motivation and satisfaction in the process of learning a musical instrument. A principal component analyses of the responsiveness and the control scales with varimax rotation showed three components for each of the underlying dimensions of *responsiveness* and *control*. The three components for *responsiveness* were a) pupil-teacher accord, b) receptiveness to parental support and c) pupil-teacher reticence. Components for the dimensions of *control* were labelled as a) pupil-teacher deference, b) pupil teacher influence, and c) pupil-parent autonomy explaining a total of about 60% of the variance. Without going further into details, the results from Creech & Hallam [6] support the hypotheses, that both positive and negative effects are involved in the process of learning a musical instrument.

Rife et al. [13] conducted one of the rare studies to examine the feelings of satisfaction by musical learning in children, on the basis of a reliable and valid measure for the assessment of private music lesson satisfaction. Therefore, a 45-item scale including positive and negative statements was administrated to *N* = 568 children between 9 to 12 years of age. Exploratory factor analyses with varimax rotation generated the unidimensional *Music Lesson Satisfaction Scale (MLSS) based on 34-items*. Internal consistency (Cronbach’s alpha = .94) and criterion-related validity was high or moderate. Additional results showed no gender differences. However, the MLSS revealed a significant effect of age. Younger children (9 years) reported higher level of satisfaction that older children (12 years). Moreover, significant differences could be found for the MLSS and the type of music instruments. Woodwind players significantly reported higher levels of satisfaction compared to string players. Rife et al. suggested, that the enjoyment of playing a musical instrument and the practicing were equally important to a child’s music lesson satisfaction. Even though the MLSS is a reliable and valid questionnaire to measure the feelings of satisfaction on musical learning, it has some issues that were important in the planning process of the present study. The most import issue was, that the Rife et al. [13] only included positive statements which leads to the fact, that negative emotions or feelings are neglected in the learning process. For example, the three highest loadings of the MLSS and the highest endorsed statements expressing intrinsic or extrinsic motivators only included positive statements such as “I like music lessons because I have a good time,” “I like that I have fun with the music I play,” “The best part of lessons is I have fun doing it,” and “I like it when I play a music piece well,” “I like when my parents say I did a good job,” “I like when my friends compliment me nicely about how I play”. However, we already know from previous studies, that learning to play a musical instrument might also include negative emotions, like experiences of failure, performance anxiety, or stress [28,30,32–34].

Recently, Mazur-Socha and Laguna [38] developed and validated an instrumental practice related affect measure (IPAM), which consists of 16 items representing four types of affect. Obviously, these comprise positive (comfort, enthusiasm) and negative (anxiety, depression) facets, indicating that this novel instrument supports the prevailing hypothesis suggesting that positive and negative facets of affect dominate the psychological impact and experiences during instrumental practice in piano students across a wide age range from children to young adults (13 to 22 years-of-age).

In sum, there appears to be consensus that varieties of positive and negative affect dominate the range of emotions perceived while learning to play a musical instrument. The present study was designed to accommodate this observation by focusing on these dimensions.

Therefore, it is of high interest for the present study, to include positive and negative items to measure the wide rate of emotions perceived while learning a musical instrument.

### Aims and research questions

This study aims to present the development and preliminary validation of a questionnaire that measures the emotional experience of children who are learning to play a musical instrument. A strong positive impact of music on emotion was reported in the studies reviewed so far [e.g. 6,13]. However, learning a musical instrument might be accompanied with strong negative emotions, as performance anxiety, pressure of learning, or even a lack of covering basic psychological needs (e.g., feeling socially included, self-endorsed or competent). To gain more insight how musical learning could be improved to keep pursuing musical activities a mostly positive emotional experience for children, an objective and time-effective measure to examine positive and negative issues is needed. Although a few questionnaires that measure musical emotions and emotion regulation in adults, adolescents and children already exist [13,36,39,40], no scale is available, that explicitly measures the positive and negative emotions in children learning a musical instrument. Therefore, we developed a questionnaire that measures positive and negative emotions in children that are learning to play a musical instrument; the *Emotions while Learning an Instrument Scale* (ELIS). Constructs included in this questionnaire are joy of learning an instrument, fear of failure as well as the influence of parents and teachers on a child`s emotions while learning a musical instrument. Particularly parental support, experiences of pressure, as well as the relationship to the teacher and to other children are considered. Results of factor analysis, item analysis, analyses of reliability and validity will be presented. To determine validity, the association with general coping as well as stress vulnerability and general mood are examined. Stress vulnerability, maladaptive coping strategies and negative general mood are expected to be related to negative musical emotions whereas healthy coping strategies and positive general mood are expected to be related to positive musical emotions. In addition, the effects of age and gender on musical emotions in children will be explored. Finally, implications for future research of musical emotional experiences in children will be discussed.

## Material and methods

### Sample

Participants were second to fourth grade students participating in a longitudinal study with three times of measurements (over 1.5 years). Students were quasi-randomly recruited from 32 elementary schools from the German federal states of Hesse, Lower Saxony and North Rhine-Westphalia. Hence, systematic school or class effects can be considered as rather unlikely. Baseline measurements were conducted at the beginning of the school year in October (T1); the second measurement was taken at the beginning of the following school year in October (T2), and final measurement was completed at the end of the same school year (T3). Only children playing an instrument are included in this study with *N*_*T1*_ = 544, *N*_*T2*_ = 400 and *N*_*T3*_ = 352 (see Table 1).

**Table 1:** Frequencies and quotas of participants’ gender, origin, and school program participation.

		T1		T2		T3	
		*N*	%	*N*	%	*N*	%
**Gende**r	*Male*	256	47.0	193	49.0	179	51.1
	*Female*	273	50.1	195	48.5	163	46.6
	*Missing*	16	2.9	33	8.3	8	2.3
**Origin**	*German*	235	43.1	188	47,2	173	49.4
	*Migrant*	247	45.3	170	42.7	139	39.7
	*Missing*	63	11.6	40	10.1	38	10.9
**Program**	*Music group*	204	37.4	189	47.5	176	50.3
	*Natural science group*	116	21.3	83	20.9	67	19.1
	*None*	71	13.0	93	23.4	97	27.7
	*Missing*	154	28.3	33	8.3	10	2.9
**Total**		**545**		**398**		**350**	

At T1 half of the sample was male, with the percentage of females declining slightly over time (see Table 1 for demographic details). Age ranged from 6 to 10 years at T1 (*M* = 7.63 years, *SD* = 0.76 years) followed by 7 to 10 (*M* = 8.53 years, *SD* = 0.68 years) and 8 to 11 years (*M* = 9.18 years, *SD* = 0.70 years) at T2 and T3 respectively. About 60% to 70% of the sample attended a school program enhancing musical (“Jedem Kind ein Instrument, JeKi” [instruments for every child]) or mathematical/scientific abilities (“Steigerung der Effizienz des mathematisch-naturwissenschaftlichen Unterrichts, SINUS” [enhancing the efficiency of mathematical and natural science classes]). Approximately 20–30% did not take part in a special school program. As information about attended school programs was not provided for 28.5% of the students at T1, percentages differ from those of T2 (12.0.% missing) and T3 (1.4% missing), when less data was missing.

### Measurement instruments

The study included nine questionnaires, scales and tests in total. Only measures relevant to the present research question will be described in the following section. Results of the other measures are reported elsewhere [41–44].

#### Development of the Emotions while Learning an Instrument Scale (ELIS)

The ELIS was designed for the assessment of positive and negative emotions in children while they are learning an instrument. As emotion regulation strategies are acquired with age [45–47] and emotional perception changes in adolescence, musical emotion in children is not expected to be as complex as it is in adolescents and adults. Therefore, a one-dimensional positive as well as a one-dimensional negative musical emotion scale was developed. A pool of 88 items were created, covering emotional engagement in learning an instrument, positive social experience, enjoyment in learning an instrument and parental support as well as stress and anxiety related to learning an instrument in different social situations (practicing alone/with other children/parents or teachers). Expert ratings led to a first selection of 23 items to enhance content validity. Since the ELIS was designed for use with children, item contents were developed with simple wording. Students answered the remaining items at three times of measurement. After T1, six items were added in order to increase reliability. Since reliability is a prerequisite for validity [48] it was considered a priority in the development of this instrument. Exploratory and confirmatory factor analysis at T2 showed a two-factor-solution. Item selection based on data at T2 led to the final version of the ELIS containing 15 items.

*Positive Emotions while Learning an Instrument* (PELI) include emotional engagement in and enjoyment of learning an instrument, a sense of progress as well as musical self-esteem, satisfaction with music lessons and parental support, as well as general well-being through musical practice (musical emotion regulation). *Negative Emotions while Learning an Instrument* (NELI) include stress due to musical practice and failure, a missing sense of progress and negative experiences regarding different social situations like playing alone, playing with other children or practicing in front of parents or teachers.

When administering the questionnaire participants are asked to mark their answers on a five-point scale ranging from 0 = *not at all true* to 4 = *completely true*. To facilitate comprehension, the scales were visualized by pictures of balloon figures of increasing size, following the example of Nigbur et al. [49].

#### Stress and coping strategies

For validation purposes, emotion regulation was measured with a shortened test inventory of stress and coping during childhood and adolescence called *“Questionnaire for the Measurement of Stress and Coping in Children and Adolescents* (Fragebogen zur Erhebung von Stress und Stressbewältigung im Kindes- und Jugendalter [SSKJ 3–8]” [50]. The SSKJ 3–8 is divided into three parts. The 84 items measure: 1. *Stress Vulnerability*, 2. *Coping Strategies* (seeking social support, problem solving, avoidant coping, palliative emotion regulation, and anger-related emotion regulation) and 3. *Psychological* (anger, sadness, anxiety) and *Physical Stress-Symptoms*. The original version of the scale assesses coping styles for two different stressful situations (academic and social). In this study, only the social situation, “fight with friend” was administered. Further, each coping strategy was reduced from 6 to 3 items per scale, as was *stress vulnerability*. Psychological symptoms were reduced from 12 to 7 items per scale. Physical symptoms were assessed by 6 items. Items were selected based on item-total correlation presented in the user’s manual. Additionally, coping via use of media devices [51] was measured with 4 items, and coping via playing a musical instrument was assessed with an additional single item designed for this study (“Imagine, you have a fight with a good friend: When something like this happens to me, I play on a musical instrument.”).

Two-week retest-reliability is high for the overall scale of coping strategies (*r* = .74—*r* = .82, (see 49). Good internal consistency is reported for the total scales (α = .79—α = .88). Reduced scales in this study showed internal consistencies of α = .60—α = .74, except for *avoidant coping* (α_*T2*_ = .39, α_*T3*_ = .58), and *anxiety* (α_*T2*_ = .52, α_*T3*_ = .47).

Convergent and divergent validity are verified as the SSKJ 3–8 showed correlations according to expectations with personality- and symptom measures as well as with coping questionnaires [50].

#### Positive and negative affectivity

To measure positive and negative affect, a German version of the Positive and Negative Affect Schedule [PANAS] [52] was used [53]. Two independent scales, *Positive Affect* (PA) and *Negative Affect* (NA), measure self-reported affectivity during different time spans. In this study, only state affectivity, not trait affectivity was assessed. NA measures subjective distress, lethargy and sadness, while PA measures enthusiasm and alertness. Response options were changed from a five-step Likert scale to a three-point scale to make answering easier for young participants. Items were reduced from 20 to 8 in T1, and to 10 in T3. In T2, the PANAS was not applied.

Adequate retest reliability as well as internal consistencies are reported for the PANAS (PA α = .86—*α* = .90, *r* = .39—*r* = .71; NA α = .84—*α* = .87, *r* = .47—*r* = .68; 51). In this study, reliability was smaller due to a shorter scale (α_*T3*_ = .61; NA *α*_*T3*_ = .53). The questionnaire shows strong relationships with other measures of emotion [54,55]. Furthermore, high correlations of PA and NA with personality traits such as extraversion and neuroticism, which are associated with negative or positive affect, have been found in children, adolescents and adults [56].

### Procedure and design

The Ethics Committee of the Faculty of Medicine of the Goethe University Frankfurt and the Ethics Committee of the Carl von Ossietzky University Oldenburg, Germany approved the study. School officials were contacted and asked whether the school wants to join the study. Teachers as well as parents were signing an informed consent. Confidentiality and anonymity of data were assured. Administration took place in classroom settings with an average of 20 students per group. For students below grade three, items were read aloud to the class by an investigator. Students then marked their answers on the answer sheet themselves.

Data was collected as part of a large-scale, longitudinal study (MEKKA) during 2009 to 2011 quantifying different cognitive and emotional variables such as concentration and coping behavior (Project number of the German Federal Ministry of Education and Research, No.01KJ0807).

### Data handling and statistical analyses

Missing data due to dropouts between the three time points were not replaced (see Table 1 for an overview of the frequencies and percentages of participants’ gender, origin, and school program participation). Data Analyses were performed using SPSS 21 and M*plus* 6 [57]. Questionnaires that had obviously been filled out in a specific pattern (e.g. all items were answered the same way) were excluded from analyses.

During data collection, new items were added between T1 and T2 due to low reliability scores, resulting in the following number of items: T1 = 23 items, T2 = 29 items, and T3 = 29 items. Our analytic strategy implied three steps. In the first step, we calculated Exploratory Factor Analyses (EFA) for T1 and T2. We applied principal axis factoring and, in order to decide on the number of factors to be retained, we analyzed the scree plot and conducted a parallel analysis [58]. In the second step, we removed weak items from the ELIS instrument based on the results of several subsequent EFAs using the T2 data and decided on the final factorial structure of the instrument. In the third step, we aimed to validate the factor structure with the final item set. We calculated the CFA for T2, and to avoid strong overfitting, we repeated the CFA for T3. When conducting the CFA, robust Maximum Likelihood estimation was used (LIT) based on the covariance matrix. For CFA, several goodness of fit indices were taken into account: **χ**^***2***^ test of model fit, Standardized Root Mean squared Residual (SRMR; [59]), Comparative Fit Index (CFI), the Tucker Lewis Index (TLI) and Root Mean Squared Error of Approximation (RMSEA; [60]). Since a significant **χ**^***2***^ test can be the result of size sensitivity [61] indices less dependent on sample size has been considered as being more reliable. Following Hu and Bentler [62] a combination of CFI or TLI values “close to.95” (p. 27) or greater and SRMR values of.08 or lower can be seen as an indication of acceptable error rates and a good data fit.

A first validation was provided by correlation analysis with related variables from SSKJ 3–8 and PANAS. The effect of age and gender will be explored by correlational analysis and *t*-test.

## Results

### Exploratory factor analysis and item selection

**Fig 1:**
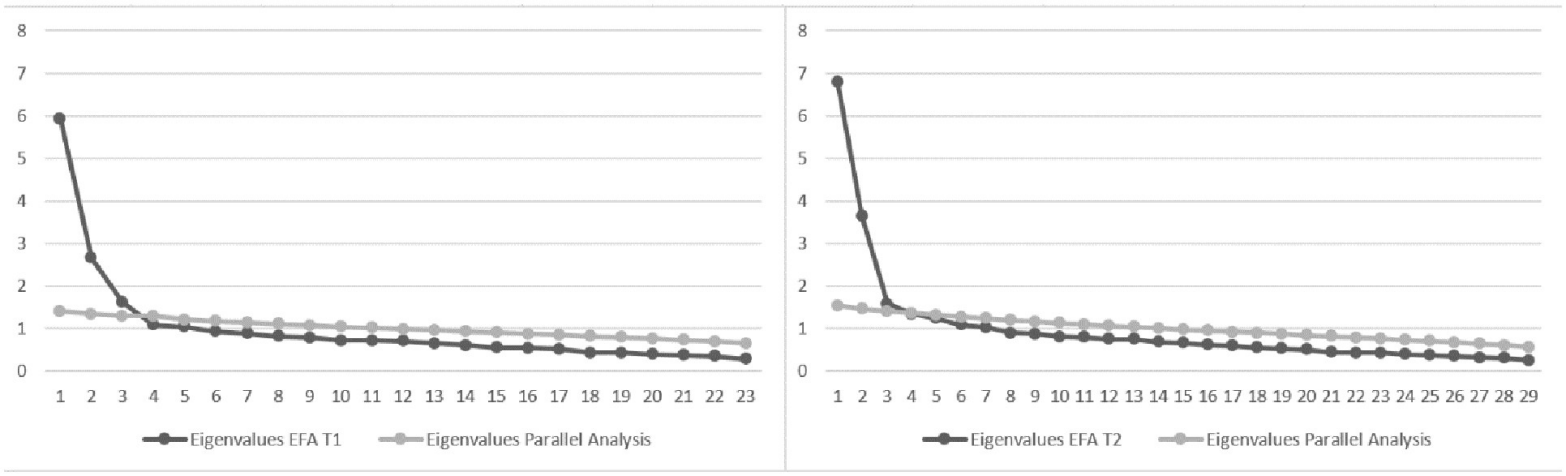
Scree plot and parallel analysis of T1 (Left) and T2 (Right) initial item pool.

In step one, a principal-axis factor analysis (PAF) was conducted at T1 and T2 (see Fig 1) to explore the dimensional structure of the items.

A scree plot analysis and a parallel analysis based in the initial principal component analysis suggested a three-factor solution at T1 (Fig 1, left). In this step, 44.3% variance were explained by these three factors with eigenvalues of 5.93, 2.66, and 1.62. The factor intercorrelations were *r*_1,2_ = -.103, *r*_1,3_ = -.463, *r*_2,3_ = .027. After the exclusion of three items due to double loadings and one item due to factor loadings < .30, the three factors’ internal consistencies (Cronbach’s alpha) were α = .85 (1^st^ factor), α = .71 (2^nd^ factor) and α = .65 (3^rd^ factor), respectively. From T1 to T2, six items were added to the original 23 items in order to increase internal consistency, resulting in a 29-item version. All items in their English translation are presented in Table 2. The items in their original German wording are listed in the S1 File.

**Table 2:** Results for the principal axis factor analysis with direct oblimin quotation at T1 and T2.

		Factors T1			Factors T2	
		1	2	3	1	2
	factor eigenvalues	5.38	1.94	4.81	5.11	2.61
	explained variance	23.37	8.45	1	21.29	10.88
**No**	**Item**					
01	I enjoy practicing my instrument.	**.55**	-.13	. 21	**.69**	-.21
02	It makes me happy that I’m getting better and better at playing my instrument.	**.80**	-.04	.13	**.55**	-.08
03	I am angry when I don’t get any further in playing my instrument.	.14	**.41**	. 06	.04	**.37**
04	I think it is nice to learn a musical instrument.	**.84**	-.09	.10	**.67**	-.13
05	My parents are proud of me when I learned something new on my instrument.	**.75**	.16	.13	**.58**	.08
06	My mother or father with me when practice.	**.34**	.04	-.13	**.32**	.11
07	It is exhausting to play on my instrument.	-.12	**.49**	. 07	-.09	**.48**
08	I’d rather do something else and practice.	.01	**.41**	. 37	**-.51**	**.38**
09	I have enough time to practice at home.	**.44**	-.02	-.12	**.45**	-.02
10	I like my music teacher.	**.64**	.01	.00	**.46**	-.02
11	When I’m happy I play my instrument.	.21	.03	**-.61**	**. 67**	.07
12	I get annoyed when others play better than me.	.01	**.51**	-.19	.09	**.64**
13	I am proud when I can play something for my parents.	**.45**	.12	-.18	**.64**	.06
14	I don’t like being my instrument in front of others.	.01	**.45**	-.00	-.16	.28
15	When I can’t play on my instrument for a long time, I start to miss it.	.20	.13	**-.48**	**.56**	.13
16	It’s embarrassing for me to play a wrong note on my instrument.	.15	**.57**	-.06	.16	**.51**
17	When I’m sad or angry I play my instrument to feel letter.	-.10	.16	**-.77**	**. 61**	.19
18	Music lessons at school are fun.	**.50**	-.11	-.16	**. 63**	-.10
19	I enjoy making music with other children.	**.50**	-. 21	-.14	**.45**	-.02
20	I feel good, when I play my instrument.	**.34**	-.02	**-.51**	**.75**	.01
21	I hate practicing.	-.10	**. 36**	**.39**	**-.57**	**.37**
22	When I practice at home, my parents are annoyed.	-.12	.26	-.03	-.08	**.27**
23	I am scared to play my instrument in front of others.	-.09	**.49**	.05	-.03	**.38**
24	I get mad when I have to practice even though I’d rather do something else.				**-.38**	**.43**
25	When I make a mistake, I’m afraid my music teacher will notice.				.11	**.59**
26	I’m sad when others play better than me.				. 07	**. 62**
27	My parents get mad when I don’t play well.				. 04	**.44**
28	I am angry when something doesn’t work doing music practice.				-.02	**.57**
29	I like playing my instrument more than I like my other hobbies.				**.48**	. 05

A second PAF of the 29 items at T2 led to a two-factor solution based on the scree plot and a parallel analysis. We decided in favor of the two-factor solution because the eigenvalue of the third factor of the PAF and the parallel analysis were almost equal. The factor eigenvalues were 6.79, and 3.60 and explained 35.94% of the variance (scree plot see Fig 1, right). Factor 1 represented children’s reports of positive emotions towards their instrument as well as positive experiences with others. The factor was named *Positive Emotions while Learning an Instrument* (PELI). Factor 2 represents items describing negative experiences associated with their instrument and the learning process, so the factor was named *Negative Emotions while Learning an Instrument* (NELI). Both factors correlated negatively, but close to zero (*r* = -.130).

In the second step, a multi-level process of item selection and factorial analysis was conducted based on several subsequent PAFs on T2-data. Data at T2 was selected instead of T1, because the latter did not contain all items of interest required for the final version of the instrument.

A first PAF (see the PAF from the step one) revealed that several items were weak indicators for the two scales of interest. Therefore, items were discarded if they showed double loadings (items 08, 21 and 24) or factor loadings < .3 (items 14 and 22). A second PAF was run to examine which effect eliminating those items had on factor loadings. Items to be excluded were determined by factor loadings < .4 (items 03, 06 and 23), resulting in a 14-item solution for PELI and a seven-item solution for NELI. To reach a more equal number of items in each scale, PELI was shortened by four items with loadings < .5 (items 09, 10, 19, and 29). After a third PAF, 2 additional items (items 17 and 15) with the lowest factor loading on PELI were excluded, resulting in a seven-item scale for NELI with α = .75 and an 8-item scale for PELI with α = .86. No items were added or selected at T3. Thus, the ELIS’s final version contains 15 items with item 1, item 2, item 4, item 5, item 11, item 13, item 18, and item 20 for the PEIL subscale, and item 7, item 12, item 25, item 26, item 27, and item 28 for the NEIL subscale (see Table 2).

### Confirmatory factor analysis

In step three of our analyses, we aimed to cross-validate our findings regarding item selection and scale construction. Therefore, we conducted a CFA on T2, and to avoid extreme overfitting, we also cross-validated the model on T3. On T2, the **χ**^***2***^ test of model fit was significant (**χ**^**2**^(89) = 212.61, *p* < .001). Based on Schermelleh-Engel, Moosbrugger and Müller [63], the **χ**^***2***^ to degrees of freedom (df) ratio still indicated an acceptable data fit (**χ**^***2***^/df = 2.1). Except for the CFI, which falls short with regard to Hu and Bentler’s [62] criteria, this model fit can be considered acceptable (CFI = .90; TLI = 0.89, SRMR = .06). RMSEA (.06) indicated an acceptable fit [64] of the two factor model on T2.

On T3, an acceptable fit was missed by a small margin (**χ**^**2**^(89) = 301.14, *p* < .001, **χ**^***2***^/df = 3.4; RMSEA = .08; CFI = .84; TLI = 0.81, SRMR = .08). By further examining modification indices to explore the misfit of this model on T3, two covariances of item pairs (items 26 and 18, items 13 and 5) turned out be significantly different from zero. Following the procedure suggested by [65] a few item modifications can be used to improve model fit, if they are practically and theoretically plausible [66]. Further, they should be well-represented by the same latent construct [67].

The wording of the items in question was: “I am sad when others play better than me” (item 26), and “Music lessons at school are fun” (item 18). In the former, the comparison of one’s skills with those of others is self-evident, but item 18, too, implies a possible comparison of one’s progress with others since it refers to group music lessons. Thus, the CFA assumption of zero correlation was very strongly satisfied here, especially because they did not appear to represent the same latent construct in this model. The second pair of items was: “I am proud when I can play something for my parents” (item 13) and “My parents are proud of me when I’ve learned something new on my instrument” (item 5), both implying information about the parents, also leading to specific error covariance that differs from a zero association.

**Fig 2:**
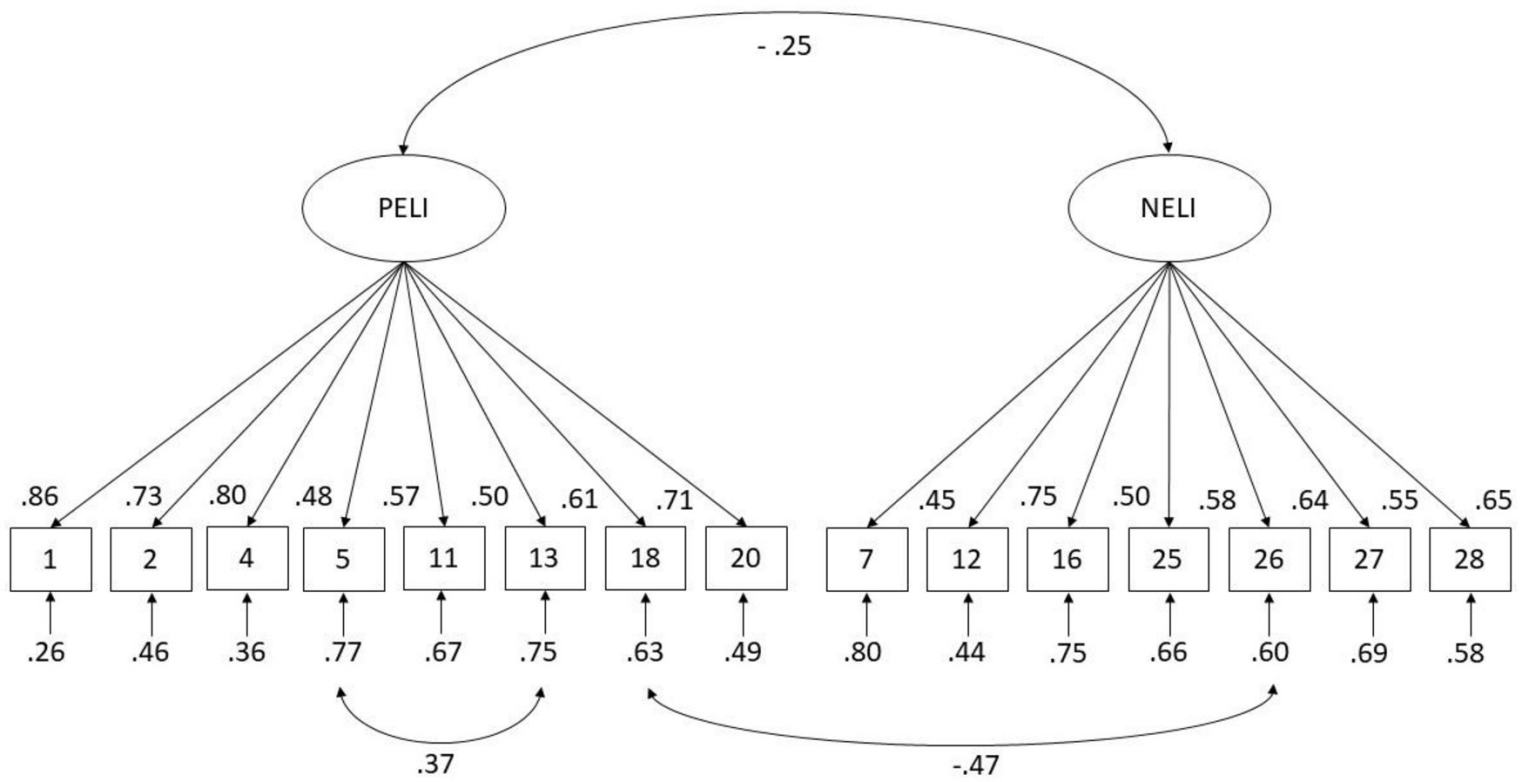
Cross validation of the factorial structure at T3 with standardized estimates.

Free estimations of error covariances of those items yielded an acceptable model fit (**χ**^**2**^(87) = 219.70, *p* < .001, **χ**^***2***^/df = 2.5; RMSEA = .07; CFI = .90; TLI = 0.88, SRMR = .07). Fig 2 details the final CFA model.

### Item statistics and reliability of the final version

At T3, the final version of PELI has item-total correlations in a mid to high range (*r*_*it*_ = .50—*r*_*it*_ = .74). NELI also shows acceptable item-total correlations (*r*_*it*_ = .37—*r*_*it*_ = .63). Item difficulty is rather high in PELI (*p*_*i*_ = 50.00 –*p*_*i*_ = 87.50), while it is low in NELI (*p*_*i*_ = 09.25 –*p*_*i*_ = 28.00). Inter-scale correlations show very small correlations of both scales (*r*_*T2*_ = -.06, *r*_*T3*_ = -.19). An overview of item statistics of the final version is shown in Table 3. Reliability of PEIL is α = .85 and ω = .86, that of NEIL is α = .78 and ω = .79.

**Table 3:** Item statistics for the final version with Item Number (No), Medium (M), Standard Deviation (SD, skewness, kurtosis, Item Difficulty (p_i_), Item Total Correlation (r_it-i_), and α when item is deleted (α_del_).

No	*M*	*SD*	*skewness*	*kurtosis*	*p*_i_	*r*_*it-i*_	α_*del*_
**PELI**							
01	3.07	1.20	-1.14	0.28	76.75	0.74	0.82
02	3.50	0.97	-2.19	4.29	87.50	0.63	0.83
04	3.34	1.05	-1.61	1.82	83.50	0.69	0.82
05	3.34	1.03	-1.64	2.07	83.50	0.50	0.84
11	2.00	1.50	-0.03	-1.41	50.00	0.53	0.84
13	3.13	1.21	-1.26	0.50	78.25	0.50	0.84
18	2.71	1.46	-0.76	-0.86	67.75	0.52	0.84
20	2.63	1.37	-0.60	-0.91	65.75	0.71	0.82
**NELI**							
07	0.97	1.21	1.02	-0.01	24.25	0.37	0.78
12	0.87	1.22	1.25	0.41	21.75	0.63	0.73
16	1.12	1.23	0.83	-0.35	28.00	0.44	0.77
25	1.09	1.42	0.99	-0.47	27.25	0.53	0.75
26	0.85	1.31	1.37	0.53	21.25	0.54	0.75
27	0.37	0.93	2.68	6.43	9.25	0.47	0.76
28	0.99	1.27	1.10	0.03	24.75	0.57	0.74

Adding items at T2 not only changed its factorial structure, it also heightened the questionnaire’s internal consistency. Reliability for NELI (α_*T2*_ = .75, *α*_*T3*_ = .78) increased in T3, while it was relatively stable for PELI (α_*T2*_ = .86, *α*_*T3*_ = .85). Retest reliabilities after 6, 12 and 18 months seem to be strong—considering the long timespan and the very young population—as can be seen in Table 4. Positive musical emotions appear to be a slightly more stable emotional process than negative emotions.

**Table 4:** Retest correlation coefficients.

	6 months T2 & T3	12 months T1 & T2	18 months T1 & T3
**PELI**	.56**	.33**	.17*
**NELI**	.42**	.21**	.11

#### Validity

*Gender and age effects*. Neither PELI (*r*_*T3*_ = -.13, *p* = .03) nor NELI (*r*_*T3*_ = .03, *p* = .57) show a relationship with age. Independent-samples *t*-tests were conducted to compare PELI and NELI scores of males and females at each time of measurement.

Even though girls (PELI *M*_*T3*_ = 3.09, *SD*_*T3*_ = 0.82; NELI *M*_*T3*_ = 0.92, *SD*_*T3*_ = 0.79) showed higher scores than boys (PELI *M*_*T3*_ = 2.74 *SD*_*T3*_ = 0.91; NELI *M*_*T3*_ = 0.87, *SD*_*T3*_ = 0.84) on both scales, only differences in PELI reached statistical significance (PELI t_*T3(df = 303)*_ = -3.54, *p* = .00; NELI t_*T3(df = 31)*_ = -0.56, *p* = .58).

*Convergent validity*. Discussing validity measures, one should bear in mind that our shortened scales (SSKJ 3–8 and PANAS) provided rather weak internal consistencies. Therefore, high correlations were not to be expected. Only significant correlations (*r* ≥.20) will be taken into account.

The correlations between the ELIS scales and the scales of the SSKJ 3–8 are presented in Table 5. PELI showed positive correlations with *Seeking Social Support* and with *Problem-oriented Coping* at both times of measurement. It also correlated to a moderate degree with an item that was added for this study to measure musical emotion regulation. PELI (*r*_*T3*_ = .34) also showed positive correlations with positive affect as measured via the PANAS.

**Table 5:** Correlations of ELIS and SSKJ subscales at each time of measurement.

	PELI		NELI	
	T2	T3	T2	T3
**Stress vulnerability**	.08	.06	.15**	**.31** **
**Coping mechanism**				
Social support	**.21** **	**.24** **	.04	.03
Problem-oriented coping	**.31** **	**.30** **	-.09	-.02
Avoidant coping	.12*	-.04	.11*	.10
Constructive palliative coping	.15**	.17**	.09	**.20** **
Destructive anger-oriented coping	.01	-.17**	.15**	**.34** **
Use of media	.01	-.04	.09	**.20** **
*Musical emotion regulation (item)*	**.42** **	**.45** **	-.03	.03
**Stress symptoms**				
Physiological symptoms	.05	-.07	**.20** **	.13*
Psychological symptoms	-.01	-.05	**.25** **	**.23** **
• Anger	-.09	-.09	**.21** **	.19**
• Sadness	.03	-.02	**.22** **	.16**
• Anxiety	.04	.00	.19**	**.23** **

Note:

**: *p* < .01;

*: *p* < .05.

NELI results were less consistent over time: At T2, NELI showed correlations *r* ≥.20 with measures of *Psychological* and *Physical Stress Symptoms*. At T3 NELI correlated ≥.20 with stress vulnerability, use of media and destructive anger-oriented coping, anxiety and negative affect as measured via the PANAS.

*Divergent validity*. PELI showed low and/or negative correlations with destructive anger-oriented emotion regulation and use of media at both times of measurement. It also barely correlated with stress symptoms and stress vulnerability, as all correlations were lower than *r* = .10 at the second and third time of measurement. Further, PELI scores were not related with NA (*r*_*T3*_ = -.01).

NELI showed small and/or negative correlations with *Social Support*, *Problem-oriented Coping* as well as with the item measuring musical emotion regulation at both times of measurement. Further, NELI barely correlated with PA (*r*_*T3*_ = .08) and showed only a small correlation with *Constructive Palliative Coping* at T3 (Table 5).

## Discussion

The present study succeeded in developing, testing, and validating a new scale of positive and negative affectivity in children learning to play a musical instrument over a period of 18 months, with tree time points. The scale is grounded in previous theoretical understanding about positive and negative emotional experiences in learning to play a musical instrument, including social aspects such as self-esteem, self-efficacy, motivation and satisfaction with music lessons, as well as parental support and pupil-teacher relationship [1,13,27]. Results confirmed the structure of the measurement model, providing evidence for the scale’s reliability and validity.

### Scale construction, factor analysis, consistency and reliability

This study presents the development and preliminary validation of the *Emotions while Learning an Instrument Scale* (ELIS). As expected, positive (PELI) and negative (NELI) musical emotions were each represented by one factor. The three-factorial solution at T1 was changed into a two-factorial structure at T2 by adding additional items to increase internal consistencies. CFA`s confirmed the two-factor model over a time of 12 months, indicating a high stability of the two- factor solution. Indices indicated an acceptable fit, except for CFI. Since a short scale often requires items to cover different constructs, item intercorrelations can be low. As the CFI implies a ratio between the target model and an independence model with the assumption of uncorrelated variables, low item-intercorrelations can lead to a small difference between both models and to a lower CFI.

As six items were selected to increase model fit on T2, cross validation of the two factorial model was examined on T3. Due to covariance of two item pairs cross validation revealed a partly acceptable fit of the two-factorial model on T3. One item pair (items 13 and 5) described parental and self-experienced feelings of pride regarding musical progress. The other item pair (items 26 and 18) described feelings of sadness and happiness in music class at school and included a (possible) comparison of one’s own progress with that of others.

Despite children’s rapid developmental changes, retest reliabilities proved musical emotion in children to be a relatively stable construct, as correlations over 6, 12 and 18 months showed. With internal consistencies of Cronbach’s Alpha α = .78, and McDonalds ω = .79 (NELI) and α = .85, and McDonalds ω = .86 (PELI) for its final version, the ELIS proves to be a reliable questionnaire that measures relatively stable musical emotions in children.

Negative musical emotions showed a small and negative correlation with positive emotions, with a slight increase over time. The small correlation is in line with results presented by Saarikallio [40] according to which musical emotions are not correlated with the expression of negative emotions through music. This would suggest that negative musical emotions and positive musical emotions, indeed, are two relatively independent constructs.

Although general positive and negative affect were first presented as two independent constructs [52], further research found them to be moderately interdependent [63,64,68,69]. Indeed, ELIS inter scale correlations seems to increase over time which could indicate a growing counterbalance between negative and positive emotional experiences. Since past research also suggests that positive musical emotions can counterbalance the stress of performing [70], further studies on the exact nature of the relationship between negative and positive musical emotions are needed.

### ELIS—Other scales and previous studies

The ELIS is the only one to measure positive and negative musical emotions in children. Its constructs overlap with other scales measuring musical experience in children, adolescents and adults.

Chin and Rickard [36] developed a questionnaire assessing quality and quantity of different forms of music use (The Music Use Questionnaire, MUSE) using two adult samples. They found five engagement styles (*cognitive and emotion regulation*, *engaged production*, *social connection*, *dance*, *physical exercise*). Two items of the PELI cover some form of musical emotion regulation. As the ELIS also includes motivational engagement in learning an instrument as well as social aspects of learning an instrument, the ELIS can be concluded to cover parts of MUSE-constructs (emotional regulation, engaged production, social connection) in a child-specific way.

Based on Saarikallio’s and Erkkilä’s [71] model of mood regulation, Saarikallio [40] developed a questionnaire for adolescents (Music in Mood Regulation, MMR) by constructing items representing the seven mood regulation strategies in their model *(entertainment*, *revival*, *strong sensation*; *diversion*, *discharge*, *mental work*, *solace)* as well as one big second-order factor of musical emotion regulation. As the ELIS covers a wide range of musical emotions in children, it does not include musical emotions that are typical for adolescents. It may therefore be necessary to develop an adolescent version of the ELIS to evaluate the development of musical emotions from childhood to adolescence. Especially since musical emotion regulation becomes more important in adolescence than in any other time of life, this might be an important addition. Like the MUSE, the MMR includes perception as well as musical production, while the ELIS is limited to children learning an instrument only. This marks the ELIS as a rather specific tool.

Creech and Hallam [1] investigated students’ perceptions of music educational settings in violin students. They adapted the 34-item Music Lesson Satisfaction Scale (MLSS; 13) measuring children`s satisfaction with instrumental music lessons, musical styles and repertoire to measure student’s enjoyment, motivation and satisfaction, their relation to their teacher, as well as parental and peer influence. They also adapted the Questionnaire of Teacher Interaction (QTI, 38) to assess how much children experience responsiveness and control cover their instrument learning. Even though ELIS does not cover all aspects of these questionnaires, its items include motivation, enjoyment, musical attainment and satisfaction with music lessons as well as parental support and peer influence.

Evans et al. [7] identified feeling a sense of progress as well as feeling self-endorsed, self-governed and socially connected by learning an instrument as important psychological needs, that need to be fulfilled to make learning an instrument a positive emotional experience for children. Interestingly, items covering a sense of self-governing (item 08 “I`d rather do something else than practice”, item 24 “I get mad when I have to practice even though I`d rather do something else”, item 25 “When I can`t play my instrument for a long time, I start to miss it”, item 29 “I like playing my instrument more than I like my other hobbies”) were excluded from the scale, as were items covering social situations other than with parents (item 10 “I like my music teacher”, item 14 “I don`t like playing my instrument in front of others”, item 14 “I don`t like playing my instrument in front of others”, item 16 “It`s embarrassing for me to play a wrong note on my instrument”, item 19 “I enjoy making music with other children”, item 23 “I`m scared to play my instrument in front of others”). This stays true even when trying to adapt the data to the three-factorial model. Even though a sense of progress and a sense of self-governing seem to have an important influence whether or not playing an instrument is continued into adulthood, neither of those constructs did emerge as a factor nor did the answers to these items fit the two (or three) factors given. As Evans et al`s [7] participants reported these psychological needs in retrospect with a timely distance of 10 years, it seems plausible that awareness of those needs is set later in live. Belonging to a group of peers, as well as feeling autonomy in one`s decisions are psychological processes typical for adolescence. As mentioned before, the ELIS does not cover musical emotions in adolescents, which again calls for an adaption of the ELIS suited for older children and adolescents to further study this topic.

### Convergent and divergent validity

Coping mechanisms are often located within two conceptual frameworks: problem-versus emotion-oriented coping [72] or approach-versus-avoidant oriented coping [73,74]. Both constructs overlap strongly: the problem-oriented coping, as well as the approach-oriented coping involve direct strategies to actively solve a situation. Emotion-oriented coping and avoidance are predominantly passive, indirect coping strategies that focus on adapting to the stressor, by avoiding it or relieving the negative emotion [50]. Although there is currently no consensus about which strategies are psychologically maladaptive, passive coping such as avoidance are generally considered to be less favorable than active coping [50,75,76].

Considering this, first indications of convergent and divergent validity seem promising. Correlational patterns between ELIS and SSKJ scales display the expected correlational structure, even though results for PELI are more consistent than for NELI. As SSKJ scales were shortened for this study, this might be due to weaker internal consistencies of the scales. Still, overall results of divergent and convergent validity seem promising.

Positive musical emotion was correlated to coping strategies generally regarded as healthy (social support, problem-oriented coping). Correlations between PELI and stress vulnerability, maladaptive emotion regulation (use of media, destructive anger, avoidant coping) and stress symptoms showed very small or no correlations, indicating PELI to be a positive emotional construct. The high correlation with the musical emotion regulation item added to the SSKJ, suggests that PELI is related to musical emotion regulation. In fact, two items could be considered measuring musical emotion regulation (item 11 “When I am happy, I play my instrument”, item 20 “I feel good, when I play my instrument”).

NELI showed significant correlations with stress vulnerability and stress symptoms which indicate that it assesses negative emotions. Correlations to constructs generally associated with psychological malfunction like destructive anger-oriented coping and use of media also show the expected correlational structures, at least at T3. Not surprisingly, the item representing musical emotion regulation within the SSKJ showed no correlation with NELI. Correlational patterns with the two PANAS subscales were also as expected, with PELI correlating moderately with Positive Affect and NELI correlating moderately with Negative Affect, establishing both scales as either positive or negative emotional constructs. Only the positive correlation of *r*_*T3*_ = .20 between NELI and constructive-palliative coping on T3 was surprising. As correlational patterns of NELI are more inconsistent than PELI`s, it leaves the question how this was influenced by weakened SSKJ scales. Further evaluating the relationship between coping mechanisms and positive as well as negative musical emotions is a challenge for further research. Furthermore, a few studies reported evidence, that learning is associated with positive and negative feelings and experiences not only in music but also in sport [77]. Further research might use a modified version of the ELIS to measure positive and negative effects of learning in sport. This would be another opportunity to validate the inventory externally.

### Age and gender

Although the gender effect was significant only for PELI, girls descriptively showed higher measures both scales. This is consistent with former studies which reported girls to experience musical emotions more intensely than boys [78].

Ratings of musical emotions did not show a significant effect of age, but it can be noted, that correlations were negative. Little is known about the development of musical emotions in children. Since participants were primarily beginners in playing a musical instrument, the intensity of experience might decrease over time, along with the novelty and excitement of playing an instrument. However, adolescents have been shown to experience the strongest musical emotions [79], and the current study covered a relatively small age range. One might therefore expect musical emotions to intensify at some time after elementary school. Whether or not a decreasing effect is typical only for elementary school children, or if it is a general phenomenon of habituation to the new learning experience that might also play a role in adolescents, requires further research.

## Limitations and implications for future research

This study has several limitations. First of all, despite the large sample size, the three time points of measurements and the demographic information gathered, there remains uncertainty about whether the quasi-experimental design of our study may diminish the conclusions derived from our results for a larger population and as reliabilities of the SSKJ 3–8 and PANAS were lower due to shortened versions some results of the correlational analysis have to be interpreted with caution. However, in educational settings, a more rigorous methodological regime may come with a cost of ecological validity. Therefore, the present approach seemed necessary for our purpose to study the positive and negative emotional effects of musical learning in real learning environments. We suggest that the large sample size, the inclusion of three time points of measurements plus demographic measures strengthen the validity and generalizability of our results.

Secondly, factorial analysis was limited to one sample. As factor structure changed from a three to a two-factor model after adding items at T2, and as cross validating the two factor model failed, it is necessary to reevaluate the factorial structure in an independent sample. Thirdly, musical emotions were only assessed in elementary school children. To evaluate its adequacy for younger and older children and to gain further knowledge of the development of musical emotions over childhood and adolescence, it would be necessary to assess negative and positive musical emotions in older and younger children. Therefore, it might be necessary to develop an adaptation of the ELIS for adolescent musicians because the wording and the range of constructs covered by the ELIS are not adequate for adolescents.

## Conclusions

To our knowledge, the ELIS is the first scale to measure positive and negative emotions in children learning an instrument. As dropout rates in Evans et al.’s [7] study showed, it is crucial to understand emotional processes and the negative impact distressful emotional experiences can have on children learning an instrument. The *Emotions while Learning an Instrument Scale* therefore not only covers a variety of constructs included in other scales, it might also help to further investigate the emotional experience of children by enabling researchers to assess positive and negative emotions in a reliable and efficient, time-saving way. Internal consistency and the first assessment of its validity have yielded acceptable to good results, indicating that the ELIS can serve as a valuable tool in assessing musical emotions in elementary school children.

## Supporting information

S1 File Original wording of the items in German language. (DOCX)

S2 File Minimal anonymized data set. (SAV)
