# Supplementary material for: Development and preliminary validation of the Emotions while Learning an Instrument Scale (ELIS)
Source: PLoS One. 2021 Aug 27;16(8):e0255019. doi: 10.1371/journal.pone.0255019 (PMC8397231; doi:10.1371/journal.pone.0255019)
Supplement: S1 File — (DOCX) [file pone.0255019.s001.docx]

**S1: Original wording of the items in German language**

|  |  |
| --- | --- |
| Nr. | Item |
| 01 | Ich habe Spaß beim Üben auf meinem Instrument. |
| 02 | Ich freue mich, wenn ich mein Instrument immer besser spielen kann. |
| 03 | Ich bin wütend, wenn ich beim Spielen auf meinem Instrument nicht weiterkomme. |
| 04 | Ich finde es schön, ein Instrument zu lernen. |
| 05 | Wenn ich etwas auf meinem Instrument gelernt habe, sind meine Eltern stolz auf mich. |
| 06 | Meine Mutter oder mein Vater sind dabei, wenn ich übe. |
| 07 | Ich finde es anstrengend, auf meinem Instrument zu spielen. |
| 08 | Ich würde lieber etwas anderes tun, anstatt zu üben. |
| 09 | Ich habe zu Hause genügend Zeit zum Üben. |
| 10 | Ich mag meine(n) Musiklehrer/Musiklehrerin. |
| 11 | Wenn ich glücklich bin, spiele ich auf meinem Instrument. |
| 12 | Es ärgert mich, wenn andere besser spielen als ich. |
| 13 | Ich bin stolz, wenn ich meinen Eltern etwas vorspielen kann. |
| 14 | Ich mag es nicht, anderen auf meinem Instrument vorzuspielen. |
| 15 | Wenn ich lange nicht auf meinem Instrument spielen kann, vermisse ich das. |
| 16 | Es ist mir peinlich, wenn ich mich auf meinem Instrument verspiele. |
| 17 | Menschen wütend oder traurig bin, spiele ich auf meinem Instrument, damit es mir besser geht. |
| 18 | Der Musikunterricht in der Schule macht mir Spaß. |
| 19 | Ich musiziere gerne mit anderen Kindern. |
| 20 | Wenn ich auf meinem Instrument spiele, fühle ich mich gut. |
| 21 | Ich hasse Üben. |
| 22 | Wenn ich Zuhause Möbel, sind meine Eltern genervt. |
| 23 | Ich habe Angst, auf meinem Instrument vorzuspielen. |
| 24 | Ich bin sauer, wenn ich üben soll, aber lieber etwas anderes machen will. |
| 25 | Wenn ich Fehler mache, habe ich Angst, dass mein Musiklehrer das merkt. |
| 26 | Ich bin traurig, wenn andere besser spielen als ich. |
| 27 | Meine Eltern werden sauer, wenn ich nicht gut spiele. |
| 28 | Ich werde wütend, wenn beim Musik üben etwas nicht klappt. |
| 29 | Auf meinem Instrument zu spielen macht mir mehr Freude als meine anderen Hobbys. |
